# Tau Protein as Therapeutic Target for Cancer? Focus on Glioblastoma

**DOI:** 10.3390/cancers14215386

**Published:** 2022-11-01

**Authors:** Rayane Hedna, Hervé Kovacic, Alessandra Pagano, Vincent Peyrot, Maxime Robin, François Devred, Gilles Breuzard

**Affiliations:** 1 Faculté des Sciences Médicales et Paramédicales, Institut de Neurophysiopathologie (INP), UMR 7051, CNRS, Aix Marseille Université, 13005 Marseille, France; 2 Faculté de Pharmacie, Institut Méditerranéen de Biodiversité et Ecologie marine et continentale (IMBE), UMR 7263, CNRS, IRD 237, Aix-Marseille Université, 13005 Marseille, France

**Keywords:** microtubule-associated protein Tau, glioblastoma, signaling pathway, medicinal chemistry

## Abstract

**Simple Summary:**

Tau protein is a microtubule-associated protein, widely known for its role in neurodegenerative diseases. Recent studies show that Tau can also be involved in the progression of many cancers as well as in cancer treatment resistance. This work overviews the role of Tau in tumorigenic processes with a focus on glioblastoma which exhibits a high expression level of this protein. Unraveling the role of Tau in glioblastoma and cancers, in general, will boost our understanding of the Tau protein and more significantly may open new avenues of strategies for curing cancer patients, in particular those with glioblastoma.

**Abstract:**

Despite being extensively studied for several decades, the microtubule-associated protein Tau has not finished revealing its secrets. For long, Tau has been known for its ability to promote microtubule assembly. A less known feature of Tau is its capability to bind to cancer-related protein kinases, suggesting a possible role of Tau in modulating microtubule-independent cellular pathways that are associated with oncogenesis. With the intention of finding new therapeutic targets for cancer, it appears essential to examine the interaction of Tau with these kinases and their consequences. This review aims at collecting the literature data supporting the relationship between Tau and cancer with a particular focus on glioblastoma tumors in which the pathological significance of Tau remains largely unexplored. We will first treat this subject from a mechanistic point of view showing the pivotal role of Tau in oncogenic processes. Then, we will discuss the involvement of Tau in dysregulating critical pathways in glioblastoma. Finally, we will outline promising strategies to target Tau protein for the therapy of glioblastoma.

## 1. Introduction

The microtubule cytoskeleton forms a functional network in eukaryotic cells. It participates in many cellular activities such as cell division, motility, morphogenesis, and intracellular trafficking of both macromolecules and organelles [1]. These processes depend on the dynamic instability of microtubules which is tightly regulated by associated proteins such as Tau. Tau is a protein that stabilizes and promotes the assembly of microtubules [2,3,4]. Interest in Tau stems from its critical role in neurodegenerative diseases, the so-called tauopathies. Indeed, the experimental models of tauopathies strongly suggest that Tau-mediated neurodegeneration results from a combination of toxic gain of function and the loss of normal Tau function [5,6,7,8,9]. Tau has also been reported to be implicated in different types of cancer. Consequently, many researchers have started to shift their attention toward understanding the role of this protein in oncogenesis [10,11,12,13]. Indeed, many studies have reported abnormal expression of Tau in breast, stomach, prostate, and brain cancer cells [14,15]. Furthermore, the expression level of Tau has been linked to resistance to anti-microtubule agents in cancer [12,16,17].

Recent advances in our understanding of Tau protein and its different localizations inside and outside neurons show that Tau has a wide interactome, including cancer-related kinase proteins. These interactions are likely to play an important role in a diverse range of cellular pathways such as cell signaling, cell motility, cellular metabolism, or genomic modifications as well as cancers, including glioblastoma, the deadliest brain tumor. Glioblastoma multiform (GBM) accounts for 52–75% of diagnosed gliomas with a median survival of patients of only 15 months [18,19,20]. Standard therapy, which consists of resection of the tumor, radiotherapy, followed by adjuvant chemotherapy with the alkylating agent temozolomide (TMZ) is unable to extend a patient’s life for more than two and a half months [21,22,23]. In addition, the efficacy of TMZ-based chemotherapy is limited in clinical application, mainly due to drug resistance [24,25]. In this context, the pathological significance of Tau protein remains poorly examined, considering that GBM tumors express variable levels of Tau [15,26]. Given the complicated nature of GBM progression, it would not be surprising that there exists a complex array of mechanisms involving Tau and diverse cellular signaling pathways where pharmacological intervention would allow opportunities to treat the disease.

Here, we review the understudied microtubule-independent functions of Tau, and its interplay with cellular signaling. Furthermore, we present Tau as a vulnerable target for cancer systems and a credible oncotarget for GBM. Moreover, we examine some of the Tau protein interactants that are initially described in neurodegenerative disorders, and which should be explored in glioma and/or GBM. Finally, we review interesting approaches to target Tau and examine efforts to apply them for GBM treatment.

## 2. Tau Structure and Regulation

**Figure 1:**
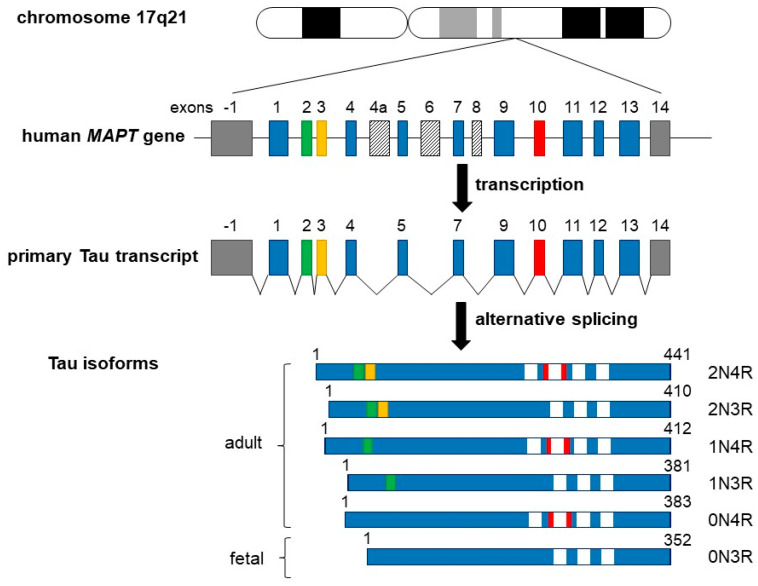
Schematic representation of the human *MAPT* (Tau) gene, the primary Tau transcript, and the six Tau protein isoforms. The *MAPT* gene is located over 100 kb of the long arm of chromosome 17 at position 17q21. It contains 16 exons (upper panel), while the tau primary transcript contains 13 exons (middle panel). Exon −1 (a part of the gene promotor) and 14 (gray boxes) are transcribed but not translated. Exons 4a, 6, and 8 (slashed boxes) are not transcribed in it. Six different Tau isoforms are shown in lower panel, which have been translated by alternatively spliced exons 2, 3, and 10, and constitutive exons 1, 4, 5, 7, 9, 11, 12, and 13, which form six different mRNAs. These isoforms vary due to the presence or absence of one or two 29 amino acids inserts, which are encoded by exon 2 (green box) and exon 3 (yellow box) in the N-terminal part in combination with either three (R1, R3, and R4) or four (R1–R4) repeat regions at carboxyl end (white boxes). The additional microtubule-binding domain is encoded by exon 10 (red box). Adult Tau includes all six Tau isoforms, including the largest isoform of 441 amino acids containing all inserts and other isoforms as indicated. The shortest 352 amino acids isoform is the only one found only in fetal brain.

In the adult human brain, there are six predominant Tau isoforms, with a primary structure consisting of 352 to 441 amino acids [27,28,29,30] (see also Figure 1 for details). Tau protein is encoded by the *MAPT* gene which comprises 16 exons encoding a single mRNA undergoing alternative splicing of exons 2–3 and 10. Tau isoforms can be distinguished by the presence of zero, one, or two N-terminal inserts of 29 residues each, involving the splicing of exons 2 and/or 3. Exon 10 can be spliced in (four repeats) or out (three repeats), encoding different microtubule-binding domain (MTBD) sequences [27,30,31,32,33,34,35]. The spliced mRNA gives therefore the six isoforms 0N3R, 0N4R, 1N3R, 1N4R, 2N3R, and 2N4R in which “N” indicates the number of N-terminal inserts and “R” represents the number of MTBD sequences. Note that the shortest Tau isoform without exons 2, 3, 4a, and 10 (also called “fetal Tau”) is predominantly expressed in the fetal brain [36,37].

Tau protein is a highly soluble and heat-stable protein and is usually considered an intrinsically disordered protein in solution [38,39,40,41,42,43,44,45]. Based on the longest 2N4R isoform, the first 44 amino acids contain a glycine-rich sequence that encompasses the two highly acidic regions (N1 and N2) [30]. Moreover, threonine (or alanine) residues are present in a higher proportion at the N-terminal region [46]. Regarding the possible function of this N-terminal region, it has been reported that Tau protein can be associated with different cell membrane proteins such as apolipoprotein A1 (preferentially with the 2N isoform) or synaptophysin (preferentially with the 0N isoform) [47], two key proteins involved in the neurite outgrowth and synaptic dysfunction [48,49].

Moreover, the N-terminal region also contains two proline-rich domains (PRD) followed by the four MTBD repeats (R1–R4) belonging to the C-terminal domain of Tau protein. Apart from their well-described involvement in binding to microtubules, the PRD regions also play a pivotal role in the interaction of Tau with diverse components of other cytoskeletons and of the plasma membrane, as we mention later [50,51,52,53,54]. Moreover, the ability of Tau to bind microtubules is mediated by its MTBD in the C-terminal region. A single MTBD is sufficient for its binding to the microtubule. Each of the MTBDs repeats has a highly homologous 18 amino acid repeat region containing a KXGS motif that can be phosphorylated [28,29,55]. Additionally, it has been shown that 3R and 4R Tau isoforms can adopt many different structures to differentially regulate their interaction with microtubules [56]. Furthermore, it has been reported that 4R Tau is more prone to phosphorylation in vitro and it is more likely to aggregate into paired helical filaments (PHFs) than the 3R Tau, a finding that could be relevant to the understanding of tauopathies such as AD [57]. In cancer, doubts remain concerning the impact of post-translational modifications such as the phosphorylation of specific residues of Tau on its function. At least, an accumulation of hyperphosphorylated Tau in the periphery of GBM tumors in patients has been reported, suggesting a possible role of Tau phosphorylation in the invasiveness of cancer cells [58]. 

Overall, tubulin, undoubtedly, remains the main ligand for the Tau protein. Under physiological conditions, up to 90% of Tau is bound to microtubules and therefore unavailable for other interactions [59]. In cancer, Tau plays a pivotal role in modifying the response to microtubules targeting chemotherapies by directly modulating microtubules and their participation in the neoplastic process [10,11,13,60]. Moreover, the binding of Tau to microtubules is very dynamic [61], which could explain why Tau can be present in intracellular compartments normally devoid of microtubules such as the nucleus. In fact, it is this presence in the nucleus that prompted and hinted to researchers that Tau has additional functions independent of its interaction with microtubules in cells. Many reports now show that Tau can co-locate and interact with multiple non-cytoskeletal proteins, many of which are involved in important cancer signaling pathways. In the following, we will explore some of these additional functions of Tau and explain how relevant they are likely to be to oncological processes.

## 3. Relationship between Tau Expression and Dysfunctions in Cancer

Tau is found with an abnormally high expression in various types of cancer such as breast cancer, ovarian cancer, gastric cancer, prostate cancer, pediatric neuroblastoma, glioma, and more [14,15,16,62,63]. Regarding these cancers, the aberrant expression of Tau is not necessarily related to the progression of this pathology. For example, high expression of Tau in several types of carcinomas, including breast cancer, prostate cancer, and gliomas [60,64,65], has been associated with improved patient prognosis. However, it seems to be related to worse survival in other cancer types such as ovarian cancer [63,66]. The question is still open regarding whether Tau expression represents a predictive marker for (neo-) adjuvant chemotherapy response or not. In vitro, Tau protein competes with microtubule-stabilizing agents such as taxane for controlling microtubule dynamic and Tau expression loss may render microtubules more vulnerable to the effects of these agents [61]. However, if we take breast cancer as an example, it appears clearly that Tau protein expression is insufficient for identifying a subset of patients with carcinomas that may benefit more from taxane-based chemotherapy [67]. For more details on the prognostic value of Tau and resistance to microtubule-targeting drugs in cancer, interested readers can refer to the review written by Papin and Paganetti [68].

Regarding its functions, cumulative studies are suggesting that the role of Tau goes far beyond its microtubule-stabilizing function and that Tau is involved in many key signaling pathways, such as cell differentiation and proliferation, morphogenesis, motility, etc. At first sight, the microtubule-stabilizing function of Tau appears relevant to cancer because the microtubule dynamic ensures several critical functions such as cell motility, cytoplasmic transport, and cell division [69]. Other functions of Tau independent of interaction with microtubules are expected to be relevant to cancer. The following paragraphs will explain each demonstrated function and how it may be associated with cancer, such as genomic instability, cell cycle, mitosis and proliferation, cell migration, and angiogenesis. 

### 3.1. Tau and Genomic Instability

One of the hallmarks of cancer is genomic instability manifesting through sequence instability and a loss of chromosomal integrity [70]. Alterations in many gene sequences confer new functional abilities for survival, proliferation, and invasiveness. Chromosomal instability can provide changes in sequence structure or the allelic number of tumor genes thus amplifying an oncogene or suppressing tumor-suppressing genes. Numerous studies brought proof that Tau can translocate to the nuclei of neurons [71,72,73,74], as well as in non-neuronal cells [75]. Using an immunofluorescence approach to label diverse tumor cells, Binder’s team reported for the first time in the 1990s the presence of Tau in the nuclei, especially near the nucleolar organizing regions of acrocentric chromosomes [76,77]. Since, it has been proposed that Tau is a DNA protector, notably by preventing stress-induced DNA breaks [78,79,80]. Indeed, the presence of Tau in the nucleus contributes to the maintenance of the DNA double helix structure and DNA winding [72,73,81]. Moreover, it has been reported that Tau functions in the nucleus are regulated by hyperphosphorylation–dephosphorylation processes, allowing Tau to bind to and protect DNA under oxidative stress conditions [45]. So, it is tempting to think that Tau could affect the progression of some cancers through a specific DNA binding site.

Other evidence that Tau is involved in chromosome integrity is the fact that specific mutations in the protein enable diverse chromosomal aberrations in cells. Rossi et al. have recently conducted a retrospective cohort study in which they analyzed cancer incidence in 15 families affected by frontotemporal lobar degeneration (FTLD) bearing *MAPT* mutations (detected by sequencing of exons 1, 9–13) [82]. Their study reveals that the FTLD family members have a significantly higher risk of developing cancer compared to control families. Moreover, bioinformatics analysis of pathways associated with the Tau protein interactome showed that the third of proteins interacting with Tau is involved in DNA damage recognition and repair, cell cycle checkpoints and phase transition, chromatin and telomere maintenance processes, and response to radiation stressors [82]. Taken together, this epidemiologic investigation supports another indirect role of Tau in DNA protection/repair systems.

### 3.2. Tau in Cell Cycle and Mitosis

The incidence of Tau in the cell cycle has been primarily explored in AD, notably to understand chromosome mis-segregation in brain cells and in peripheral tissues [83,84,85,86]. Physiologically, neurons charged with neurofibrillary tangles (NFT) hyperphosphorylated Tau undergo an abnormal cell cycle entry before apoptosis [87,88]. In the case of cancer, it is surprising that the impact of Tau phosphorylation on mitosis has been poorly examined considering the significant proliferative activity of tumor cells. In this regard, a recent study focused on the effect of Tau on mitosis: Flores-Rodriguez et al. have shown that the quantity of Tau phosphorylated on Thr231 in the PRD region (pT231-Tau) correlates to phases of cell division in the SH-S5Y neuroblastoma cell model [89]. Indeed, the authors at first observed an increased amount of pT231-Tau concomitantly with the placement of mitotic spindles and condensation of chromosomes during prophase and metaphase up to fill the entire cellular cytoplasm. Then, the quantity of pT231-Tau decreased markedly to diffuse granular staining during anaphase and telophase. These results suggest that the role of pT231-Tau is to maintain the integrity of the DNA and chromosomes during mitosis. In addition, the presence of Tau protein, maybe unphosphorylated here, could be involved in the separation of sister chromatids. To clarify, Bougé and Parmentier have recently reported the effect of Tau expression on chromosome mis-segregation in vivo [90]. Using models of wing disc epithelium of *Drosophilia* and tumor HeLa cells, the authors show that an excess of Tau induces mitotic arrest, with the presence of monopolar spindles. Interestingly, they also demonstrated that the mitotic defect occurs through the inhibition of the kinesin Klp61F, the *Drosophilia* homolog of Kif11 which ensures chromatid transport onto spindles. In cancer, it is hence questionable whether Tau could play a role in carcinogenesis or tumor progression, notably by defecting programmed cell death, necrosis, or senescence [91].

Moreover, many microtubule destabilizing and stabilizing compounds, called microtubule targeting agents (MTA) such as vinca alkaloid or taxoid, have been widely used as antimitotic agents for their induction of cell cycle arrest and apoptosis [92,93]. However, the relationship between the expression of Tau observed in many cancers and the relapse of patients who have followed a therapeutic protocol involving MTA is still conflicted [12,94]. One hypothesis may imply MTA-mediated activation of different cell death pathways such as autophagy, a mechanism in which the quantity of Tau could promote. Indeed, the potential association between autophagy inhibition and Tau has been recently investigated in prostate cancer cells where the presence of aberrant monoastral mitotic spindles has been evidenced to correlate both with the accumulation of Tau oligomers and with a recovered cytotoxic effect of docetaxel [95]. Interestingly, the authors suggest that cancer could maintain a high Tau protein turnover to avoid the formation of toxic oligomers, mainly during mitosis, through defective autophagy. Therefore, interfering with Tau turnover could represent an effective strategy to counteract the resistance to antimitotic agents such as MTA.

### 3.3. Tau in Cell Migration

Another characteristic of cancer is exacerbated cell migration. This process involves strong cell shape modifications and thus heavily relies on the redistribution and cooperation of cytoskeletal filamentous proteins, notably actin filaments and microtubules. While the requirement of crosstalk between microtubules and actin is not questioned, mechanisms underlying the microtubule-actin organization by cross-linkers remain largely unexplored, and little is known about the functional contribution of Tau protein. The impact of Tau overexpression on cell motility was investigated by Morris and coll. using an IDH wild-type human GBM-derived LN229 cell line [96]. The authors showed that high Tau expression selectively reduced cell migration without affecting cellular proliferation or apoptosis. As a reminder, isocitrate dehydrogenases (IDH) are enzymes that catalyze the oxidative decarboxylation of carbohydrates in the Krebs cycle to produce ATP in the mitochondria [97].

**Figure 2:**
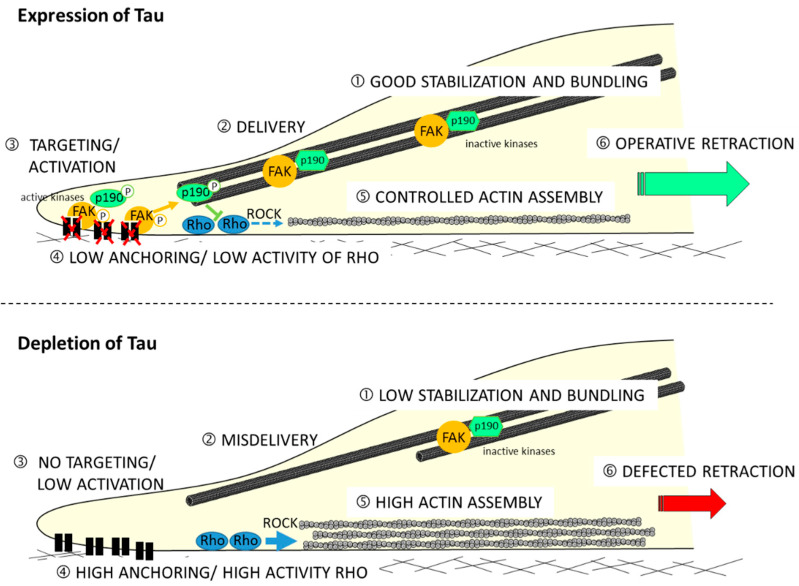
Tau regulates the microtubule-dependent migration of glioblastoma U87MG cells via the Rho-ROCK signaling pathway. Directional cell migration requires spatiotemporal control of focal adhesion formation and dissociation: (upper model) in Tau-expressing cell, good stabilization and bundling of microtubules (1) are positioned at the tail of polarized cells; during migration, the active phosphorylated forms of FAK and p190-RhoGAP proteins are delivered (2) to focal adhesion at the rear of cells (3), thus reducing anchoring and limiting activity of the Rho-ROCK axis (4); this could moderate actin polymerization (5); and good tail retraction results on, which contributes to a coordinated back-to-front movement of cell; (lower schema) in Tau-depleted cells, microtubules are less stabilized and weakly organized in bundles in the back of cells (1); the delivery of FAK and p190-RhoGAP proteins to the back of cells is defected (3); high anchoring on focal adhesion and removal of the restrictive activity of the Rho-ROCK pathways (4) leads to excessive actin polymerization (5); and bad tail retraction is observed.

Moreover, the mechanism showing how Tau protein is involved in the migration process is not described. Recently, by using the shRNA approach to deplete Tau in GBM-derived U87MG cells (shTau cells), the authors determined its impact on cytoskeletal coordination during migration [98]. Results have revealed a 36% reduced motility in the shTau cells compared to the control cells with no impact on directionality. By analyzing the spatial organization of microtubule and actin networks, they observed extensive actin cables but limited microtubule bundling at the back of polarized shTau cells. This remodeling of cytoskeletons resulted in weak coordination between protrusion at the leading edge and retraction of the trailing edge of shTau cells. Authors also demonstrated that the remodeling of the actin cytoskeleton depends on the relocation of Rho-associated protein kinase 1 (ROCK1), as well as regulating kinases p190-RhoGAP and FAK. They therefore propose the following model (see Figure 2).

In addition, it is worth mentioning that Tau can also directly bind to actin as proven by many studies [99,100,101,102,103]. In this regard, Tau exhibits a similar binding mode to microtubules and actin filaments. Indeed, using reconstituted high-viscosity networks of microtubules and actin filaments from purified tubulin, actin, and Tau, Farias et al. reported that Tau can interact with each polymer individually by MTBD regions [104]. Moreover, the ability of Tau to bundle and crosslink actin is regulated by phosphorylation at its serine and tyrosine residues in the PRD region [105,106,107]. As mentioned above, studies by He et al. demonstrated, with Tau mutants having the PRD region but lacking the MTBD region, that Tau can induce the assembly of monomeric actin to filamentous actin polymers and promote F-actin bundling [53]. Thus, the way in which Tau governs the crosstalk between microtubules and actin cytoskeletons remains an unsolved puzzle, with the latest studies showing that both MTBD and PRD regions of Tau are important for their interaction with actin.

### 3.4. Tau and Angiogenesis

Angiogenesis is defined as the formation of new blood vessels from pre-existing ones. This process involves the migration, proliferation, and differentiation of endothelial cells, which line the inside wall of blood vessels. The involvement of Tau has been mainly explored in tauopathies including AD for which we can assist in active neoangiogenesis [108,109,110,111,112,113,114]. To better understand the role played by Tau in this process, Bennett and coll. made use of the tetracycline-repressible gene expression cassette to deplete P301L-mutated Tau in aged rTg4510 mice [115]. Although Tau expression neither affected vascular remodeling nor vascular barrier functions, the authors evidenced that many genes involved in endothelial senescence and in recruiting leukocytes to the endothelium were upregulated in the microvessels (including, i.e., *Serpine1*, *IL8*, *ICAM-2* and *TIE1*). Altogether, these findings show that pathological Tau deteriorates brain microvasculature and increases the expression of senescence genes in endothelial cells, which may contribute to the impairment of cerebral blood flow in AD. 

Excessive abnormal angiogenesis plays a crucial role in cancer, particularly for solid tumors in which there is an ever-greater nutrient and oxygen demand [116]. Neoangiogenesis triggered by tissue hypoxia and the progression of neoplastic lesions occurs in several key steps [117,118,119,120]. The initially avascular tumor cell mass, upon reaching a critical size, cannot ensure its further growth. Hypoxic cells of the tumor mass, via the secretion of proangiogenic factors such as vascular endothelial growth factor (VEGF), Angiopoietin-1 and 2 (Ang1/2), or platelet-derived growth factor (PDGF), initiate angiogenic switch by stimulating peripheral endothelial cells of parenchymal tissue microvasculature. To our knowledge, the only study showing the involvement of Tau in cancer angiogenesis is the collaborative work led by Prof. Sanchez-Gomez on IDH mutant gliomas [65]. Here, the authors first showed that the expression of Tau depends on the genetic status of IDH1/2 and that it hinders the progression of the tumors. Then and more interestingly, they found that Tau protein inhibits angiogenesis and favors vascular normalization in gliomas expressing wild-type EGFR. The mechanism behind this inhibition is shown to be a result of Tau’s opposition to the EGFR–NF-κB–TAZ signaling pathway which favors the secretion of vascular components (αSMA and CD248). By blocking this signaling pathway, Tau could impede the plasticity of the tumor cells and their capacity to generate mesenchymal-pericyte-like cells necessary for tumor progression. However, an aberrant vasculature is observed when Tau or when EGFR are mutated. The relationship between Tau and EGFR proteins will be further examined when we discuss the RTK-Ras/MAPK/ERK signaling pathway.

## 4. Tau in Glioblastoma

A limited understanding of the underlying biology of GBM contributes to the lack of clinical advances in its therapy. For two decades, many studies were conducted to establish gene expression profiles of GBMs which permitted its grouping into several subclasses reflecting patients’ survival outcomes [121,122,123]. In 2010, Verhaak et al. analyzed The Cancer Genome Atlas (TCGA) Research Network data [124] and identified four molecular subtypes of GBMs based on distinct gene expression signatures reflecting the cell types of origin: classical (from astrocytic cells), mesenchymal (from astroglial cells), proneural (from oligodendrocytes), and neural (from neural cells) [123]. Recently, Berman’s team examined 543 cases of GBM from the TCGA, and the authors were able to shed light on core pathway alterations responsible for the occurrence and the progression of gliomas: EGFR, IDH1, NF1, PDGFRA, PIK3R1, PIK3CA, PTEN, RB1, and p53 [125]. The fact that Tau can be a ligand of many kinases and transcription factors drives us to discuss their impact on GBM progression. Table 1 below will overview Tau’s interactions with partner signaling pathways and their effects on cellular responses. 

### 4.1. Tau and Altered Pathways in Glioblastoma

#### 4.1.1. The Receptor Tyrosine Kinases Signaling Pathways

A large group of genes in all eukaryotes encodes for membrane-spanning surface receptors, among them we have one large family supplying intrinsic protein tyrosine kinase activity, the receptor tyrosine kinases (RTKs). RTKs regulate proliferation, survival, differentiation, migration, and angiogenesis through the activation of two major downstream pathways Ras/MAPK/ERK and PI3K/AKT. The function of RTKs is to catalyze the transfer of the γ phosphate of ATP to hydroxyl groups of tyrosine residues on target proteins which serve as docking sites for cytoplasmic signaling effectors. Numerous reviews focus on six major tyrosine kinase receptors; the epidermal growth factor receptor (EGFR), the vascular endothelial growth factor receptor (VEGFR), the platelet-derived growth factor receptor (PDGFR), the hepatocyte growth factor receptor (HGFR/c-MET), the fibroblast growth factor receptor (FGFR) and the insulin-like growth factor 1 receptor (IGF-1R) (see also well-described reviews [147,148,149]). Alteration of these receptors is commonly observed and at least one RTK was found altered in 67.3% of GBM; the EGFR in 57.4% of GBM, PDGFR in 13.1% of GBM, HGFR/c-MET in 1.6% of GBM and FGFR in 3.2% of GBM [125]. Additionally, the RTK/Ras/MAPK/ERK and RTK/PI3K/AKT pathways were found to be altered in more than eight out of ten patients [125]. However, the importance of the Ras protein and downstream protein signaling remains unclear to the pathogenicity of GBM. Apart from GBM, Makino and coll. recently conducted a multiplexed sequencing on 242 glioma tumors for mutations of *IDH1/2*, *H3F3A*, *HIST1H3B,* and *TERT* genes concomitantly to *RAS* isotypes [150]. Overall, they found very little correlation between *RAS* mutations and these other genes, as well as no clear association, was identified between *RAS* mutations and the histological phenotype of glioma. The accurate diagnosis of GBM involving Ras signaling needs further discussion.

##### Tau and the RTK/Ras/MAPK/ERK Pathway

Mitogen-activated protein kinases (MAPKs) are highly conserved serine/threonine protein kinases that could be directly activated by extracellular stimuli, typically including oxidative stress, growth factors, and pro-inflammatory factors [151,152]. MAPK includes ERK1/2, p38, and JNK. They can induce many cellular responses, such as proliferation, invasiveness, differentiation, and autophagy [153,154]. The role of abnormal activation of the RTK/Ras/MAPK/ERK pathway through genetic alterations in cancer has been extensively investigated [151]. In GBM, the specific gain-of-function mutation of EGFR, and more specifically the constitutive activation of EGRF variant type III (EGFRvIII), is considered as a driver mutation to activate the MAPK/ERK pathway which consequently leads to tumorigenesis [155,156,157].

Gargini et al. recently characterized the effects of the high expression of Tau protein in a subset of gliomas, especially in IDH mutant tumors, where it seemed to impair tumor aggressiveness [65]. Based on in silico gene analysis, it was found that the EGFR/MAPK pathway was co-upregulated with Tau. Using mice orthotopically injected with RG1 cells expressing the IDH1 gene or its inactive variant IDH1R132H, the authors also showed that IDH1R132H tumors were less aggressive and expressed more Tau protein in comparison to IDH wild-type tumors. More interestingly, they also revealed an inverse correlation between the Tau levels and phosphorylated EGFR in tumors. Note that no difference was observed for mice developing tumors expressing EGFRvIII and/or Tau protein. Thus, these results strongly suggest a negative effect of IDH and Tau on the EGFR/MAPK/ERK pathway. On downstream of the signaling pathway, the authors demonstrated that Tau blocked the mesenchymalization of EGFR glioma cells by inhibiting NF-κB phosphorylation and by reducing the amount of TAZ protein. Altogether, their results indicated that Tau expression can induce changes in the glioma phenotype, through the regulation of the EGFR/TAZ/NF-κB pathway. However, once again, the transposition of these conclusions from gliomas to GBM cannot be completed so simply, as these two models present numerous histopathological, genotypic, and protein differences.

##### Tau and the RTK/PI3K/AKT Pathway

The RTK/PI3K/AKT pathway is activated by transmembrane tyrosine kinase growth factor receptors, transmembrane integrins, and G-protein-coupled receptors. Upon activation of these receptors, the phosphoinositide 3 kinase (PI3K) translocates to the plasma membrane and produces phosphatidylinositol 3,4,5-triphosphate (PIP_3_) from phosphatidylinositol bisphosphate (PIP_2_) [158,159,160]. A cascade of phosphorylation follows, first for AKT (at threonine 308 and serine 473), then the downstream effectors of PI3K/AKT signaling, mTOR kinase. Moreover, AKT indirectly inhibits apoptosis by phosphorylating and inactivating the pro-apoptotic protein GSK3 [161]. Interestingly, Tau is one of the well-described targets of mTOR and GSK3 [162,163]. 

**Figure 3:**
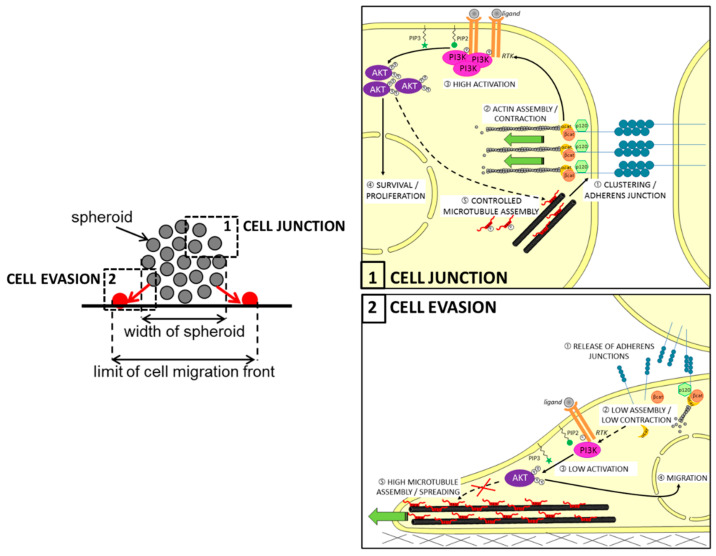
Proposed model for the role of Tau in regulating multicellular spheroid organization, growth, and migration via the PI3K-AKT signaling pathway in glioblastoma. Upper left panel: principle of cell evasion and cell junction in spheroid: in the zone (1), the N-cadherin junctions maintain cell-cell adhesion necessary for the wholeness of spheroid; in the zone (2), cells closed to the support leave spheroid to migrate individually (in red). Upper right panel: in the context of PTEN-null mutated cells, Tau could contribute by microtubule-dependent function to (1) stabilize the N-cadherin/β-catenin complexes in clusters to reinforce cell–cell adherens and to (2) promote the actin assembly required for cell contraction; Tau promotes indirectly activations cascade of the RTK/PI3K-AKT signaling pathway (3), resulting in survival and proliferation of cells (4); also, active AKT may control the microtubule assembly by increasing Tau phosphorylation (5). Lower left panel: following the adhesion of cells to the support, the release of adherens junctions further promotes cell detachment from spheroid (1); this results in reduced assembly of actin and weak contraction of the cell (2), then low activation of the RTK/PI3K/AKT pathway (3); active AKT is regulating genes involved in cell migration (4), as well as Tau-promoted assembly of microtubules contributes to cell spreading. β-cat: β-catenin; α-cat: α-catenin; PIP2: phosphatidyl-inositol 4,5; PIP3: phosphatidylinositol-3,4,5-trisphosphate.

It has been previously reported that Tau can interact with some of the components of the RTK/PI3K/AKT pathway. For example, several in vitro studies showed that Tau can associate with the regulatory subunit p85 of PI3K through the SH3 domain of the subunit [14,128]. Although the consequences were not explored here, it is tempting to think that direct Tau-to-PI3K interaction places Tau in the pathway upstream, as previously described for prostate cancer [127]. In this regard, Pagano et al. recently conducted in silico analysis of reverse phase protein arrays (RPPA) data from a cohort of GBM patients (TCGA-GBM dataset). They found a significantly lower level of activated AKT, notably phosphorylated on serine 473 (pAKTSer473) in human GBM expressing low levels of Tau RNA (*MAPT* gene, with *p*-value = 0.0078). The authors confirmed this result both by performing a PathScan^®^ intracellular signaling array and then by immunoblotting on cell lysates. Moreover, treatment of Tau-expressing U87MG cells by the PI3K inhibitor LY294002 significantly reduced the growth of multi-cellular spheroids (MCS), while the AKT inhibitor perifosine mainly disrupted their evasion. As expected, both growths of MCSs and evasions of Tau-depleted cells from MCS were not significantly affected by the two treatments. More interestingly, the depletion of Tau reduced paracellular permeability into MCS originating from the mislocation of the N-cadherin protein at the cell membrane. Altogether, these findings showed for the first time that Tau is an upstream regulator of the PI3K/AKT signaling, likely through the remodeling of microtubules favoring the correct delivery of N-cadherin into the membrane; hence, the AKT activity is necessary for Tau-dependent cell migration during the EMT, while the upstream PI3K activity is rather involved in the control of growth and survival of GBM cell tumors (see also Figure 3).

##### Tau and PTEN to Regulate the RTK/PI3K/AKT Pathway

The tumor suppressor gene *PTEN* (phosphatase and tensin homolog deleted on chromosome 10) is the second most frequently mutated gene after *P53* in many human sporadic and hereditary cancers [164,165]. PTEN antagonizes the PI3K/AKT pathway by dephosphorylating PIP_3_ to PIP_2_. The lipid phosphatase activity of PTEN is critical for its tumor-suppressor function. Indeed, it has been reported that PTEN-null mice die at early embryonic stages, and heterozygous *PTEN* knockout mice develop numerous tumors [166,167,168].

The role of Tau protein in the PTEN/PI3K/AKT pathway has been poorly explored in cancers [169,170,171], and at most it has been reported that Tau expression is inversely correlated to *PTEN* mutation/deletion in various cancers including gliomas [65]. In GBM, *PTEN* mutation or deletion is observed in more than 40% of patients, making it one of the most frequent genomic events of the disease [164,172,173,174,175]. Its dominant-negative effect is seen in the constitutive activation of the PI3K/AKT signaling pathway. In this context, recent results on PTEN-deleted U87MG cells showed that the depletion of Tau was associated with reduced AKT activation, as demonstrated with low pAKTSer473 [129]. Again, Pagano et al. also found a positive association between pAKTSer473 and *MAPT* RNA expression in the publicly available TCGA human GBM datasets, suggesting that Tau contributes to AKT activation in a context of a loss of function of PTEN. In agreement with these results, a recent publication revealed a positive association between increased phosphorylation of AKT on Ser473 in patient tissue samples and progression from anaplastic astrocytoma to GBM [130]. Despite all these observations, the mechanism of action relating to Tau and PTEN remains unidentified. In opposition, the relationship between Tau and PTEN has been extensively examined in AD pathogenesis [176,177,178,179,180]. Indeed, it has been observed that a significant loss of PTEN correlates with a dramatic increase in the concentration of phosphorylated Tau at Ser214 in the PRD region in NFTs [179]. Mechanistically, PTEN downregulates AKT, which in turn activates the Glycogen synthase kinase 3 beta (GSK3β) phosphorylating Tau at multiple sites in vitro and in vivo [181,182,183,184]. Altogether, it would be interesting to examine if the pAKTSer473-Tau-PTEN triad may define a prognostic marker of poor GBM outcome.

#### 4.1.2. The Src Family Kinases Signaling Pathways

*Src* designates a family of proto-oncogenes encoding a family of non-receptor tyrosine kinases, called Src family kinases (SFK) (see [185,186,187] for details). c-Src was the first protein kinase to be described as capable of phosphorylating tyrosine residues [188]. SFKs interact with multiple cell surface receptors including integrin and RTKs such as EGFR, PDGFR, and VEGFR [189]. They are activated rapidly upon receptor activation, and they participate in the transduction and regulation of signaling events involving cell adhesion, migration, invasion, proliferation, apoptosis, and angiogenesis [185]. Src has been quite well-explored in the context of cancer, notably for its impact on PI3K/Akt/mTOR, MAPK, and PDGF signaling pathways [190,191]. In addition, it has been reported that six members of SFKs (Fyn, c-Src, Yes, Lyn, Lck, Hck) are expressed in gliomas and actively contribute to the malignancy of tumors [192,193,194,195,196,197]. The following section will talk about the role of two major SFKs, Src and Fyn, in GBM, and will focus especially on their interaction with Tau.

##### Tau and Src Protein

In GBM, Src protein is neither overexpressed nor mutated but it is highly hyperactivated compared to normal brain tissue leading to the aberrant transduction of its signaling cascades [124,194]. In addition, the role of Src in GBM invasion is demonstrated in transgenic mouse models where GBM invasion was significantly decreased in *Src* knockout mice compared with control mice, suggesting that Src expression by normal brain tissue is required for invasion and infiltration of GBM cells [198]. Moreover, the hyperactivation of Src in GBM also contributes to processes such as inflammation and metabolism, which help in the establishment of the tumor microenvironment and its development [199]. Overall, these data could indicate that the hyperactivation of Src is mainly a consequence of the aberrant activity of other signaling pathways, i.e., RTKs as suggested by [200,201].

As with all SFKs, Src consists of SH domains that include the SH3 domain. This domain can recognize the proline-rich region of proteins such as the PXXP motif of the PRD region of Tau, as it has already been demonstrated experimentally both in vitro and in cells [54,202]. A potential consequence of the SH3 interaction is the upregulation of tyrosine kinase activity. Indeed, Lee’s team demonstrated in PDGF-stimulated fibroblasts that Tau appeared to prime Src for activation following PDGF stimulation, as reflected by changes in Src-mediated actin rearrangements [99]. In addition, Tau-expressing cells showed sustained actin breakdown, even in the absence of PDGF stimulation, unlike cells that lacked Tau protein. These observations indicate that the interaction of Tau with Src may serve as a mechanism for coupling extracellular signals to the actin cytoskeletal system. Interestingly, the authors have demonstrated that microtubule association by Tau was not required for the observed changes in actin morphology. These findings are interesting because they suggest that targeting Tau can mediate the function of the protein, and thus it can give a prognostic advantage for GBM patients.

##### Tau and Fyn Protein

In GBM, Fyn was found to be overexpressed in patient samples relative to both normal brain and all other tumors, with varying patterns of expression [192,203]. This kinase has also been shown to be downstream of the commonly mutated RTKs in GBM, such as EGFR, PDGFR, and c-MET [144]. In addition, genetic and pharmacologic inhibitions of Fyn block EGFR-dependent motility and tumor growth both in vitro and in vivo [192]. Recently, Lowenstein’s team evaluated the role of Fyn using genetically engineered glioma mice models (GEMMs) [204]. The team clearly demonstrated that *Fyn* knockdown reduced tumor progression and significantly increased survival in diverse immune-competent GEMMs of glioma. More interestingly, examination of glioma immune infiltrates displayed a reduction in the amount and in the activity of immune suppressive myeloid-derived cells in the Fyn glioma tumor microenvironment. These findings suggest that targeting Fyn in GBM may help both in reducing tumor cell proliferation and in making the immune microenvironment more engaged in tumor striking. 

Interestingly, Tau protein also interacts with Fyn through the SH3 domain. Curiously, little or no study was interested in the relationship between Fyn and Tau in a cancer context. In the 1990s, many studies demonstrated that the pattern of Fyn expression in the brain correlates well with that of Tau and that the two proteins are localized in the growth cones of neurons [145,146,205,206,207]. They are also expressed in oligodendrocytes where they participate in myelinogenesis [208,209]. For these reasons, it would be interesting to further explore the role of Tau and Fyn in GBM tumorigenesis.

#### 4.1.3. The p53 Signaling Pathway

The transcription factor, p53, is critical for many important cellular functions involving genome integrity, such as cell cycle control, DNA damage response, and apoptosis. The p53 protein is encoded by the *TP53* gene located on chromosome 17p13.1. In cancer, p53 is considered a broad suppressor factor suppressing over 2500 genes involved in tumorigenesis and tumor invasion. Under normal physiological conditions, and in response to diverse stress signals such as DNA damage, p53 induces DNA repair, cell cycle arrest, and cell apoptosis. Thus, an inactivating mutation in the *TP53* gene or its negative regulation results in tumorigenesis. Most GBM patients (about 87% of cases) and cell models (about 94%) exhibit dysregulation of the p53/ARF/Mdm2 pathway [124,210]. Based on multidimensional and comprehensive characterization of more than 500 GBMs, Brennan and coll. described the landscape of somatic genomic alterations in which mutation or deletion of *TP53* is identified in about 28% of cases, amplification of the negative p53 regulator Mdm2/4 in 15%, and/or deletion or mutation of its own negative regulator CDKN2A in 58% of patients [125]. Moreover, dysregulated p53 pathway components contribute to cell invasion, migration, proliferation, evasion of apoptosis, and cancer cell stemness of GBM (see the well-detailed review [211]).

The relationship between Tau and p53 has been mainly explored in neurodegenerative diseases [212,213,214,215]. Indeed, it has been observed that the levels of p53 are increased in the AD brain, which is also accompanied by an accumulation of hyperphosphorylated Tau (see review [216]). Meanwhile, no direct relationship between Tau and p53 has so far been clearly demonstrated in GBM. However, Tau is necessary to transport p53 into the nucleus in response to DNA damage. In this context, Giannakakou and coll. demonstrated in numerous tumor cell lines that the trafficking of both wild-type and mutant p53 was granted by a functional microtubule network since cell treatment with microtubule-disrupting agents, vincristine, and taxol, drastically reduced nuclear accumulation of p53 [132]. Another way, through which Tau may be implicated in this pathway is by modulating p53 and/or other p53-pathway components. To support this statement, we can cite a recent study demonstrating the effects of Tau on the p53 function [133]. The authors showed in the neuroblastoma cell model that Tau downregulation impacted p53 stability which affected cell fate by increasing cellular senescence and by decreasing apoptosis. They also showed that Tau modulates the DNA damage response by post-translational modification of p53 and Mdm2 [133].

Altogether, it is tempting to think that Tau could be involved in the suppressive role of p53, both directly and indirectly most likely due to its microtubule-stabilizing function. In addition, the Tau protein can interact with many other protein kinases with significant roles in gliomagenesis. The following chapters list some of these proteins and their roles in GBM.

### 4.2. Other Altered Kinase Activity Characterized in Tauopathies

#### 4.2.1. The Glycogen Synthase Kinase 3 Signaling Pathway

Glycogen synthase kinase-3 (GSK3) is an unusual serine/threonine kinase involved in diverse cellular functions ranging from glucose metabolism and energy homeostasis to proliferation and apoptosis [217]. GSK3 is considered of some interest because it has some unconventional characteristics for a kinase: being constitutively active, its substrates usually need to be pre-phosphorylated by another kinase, and it is inhibited, rather than activated, in response to stimulation of the two main signaling pathways known to impinge on GSK3, (1) the PI3K/AKT-dependent pathway that is triggered by insulin and growth factors, and (2) the Wnt signaling pathway that is required for embryonic development. Major findings focus on the fact that GSK3, particularly the beta isoform of GSK3 (GSK3β), is a key kinase contributing to abnormal phosphorylation of Tau in NFTs in AD [218,219,220].

Multiple studies have tried to identify the role of this kinase in the glioma and GBM progression [134,221,222,223], and its relationship with Tau can so far only be assumed by circumstantial evidence. In fact, GSK3β could impact Tau via 14-3-3 proteins which constitute a family of highly conserved and ubiquitously expressed small phosphoserine-binding proteins [224]. This family of proteins has seven members which can interact with multiple phospho-proteins. The isoform ζ was reported to be overexpressed and to play a role in GBM tumorigenesis [135,136]. Interestingly, 14-3-3 ζ mediates Tau hyperphosphorylation by multiple kinases [137,138], including active S9-phosphorylated GSK3β kinase. Mechanistically, 14-3-3 ζ promotes the stabilization of the Tau-GSK3β protein complex [139,140]. Another way by which 14-3-3 could interfere with Tau was reported by Joo and coll. who evidenced that overexpression of the σ isoform promotes Tau detachment from microtubules in neurons [225]. Overall, we should further examine how GSK3β/14-3-3 could contribute to GBM tumorigenesis by acting on the microtubule-stabilizing function of Tau.

#### 4.2.2. The Cyclin-Dependent Kinase 5 Signaling Pathway

Cyclin-dependent kinases (CDKs) are a family of serine/threonine protein kinases first discovered for their role in regulating the cell cycle, but are also involved in regulating transcription, mRNA processing, and the differentiation of nerve cells (see book of [226]). CDKs require cyclins for their activity as cell cycle regulators in proliferating cells.

CDK5, however, is an unconventional member, because (i) it does not participate in cell cycle progression, and (ii) it is activated by the binding of its special regulatory subunits p35 or p39 instead of cyclin like the other CDKs. Indeed, CDK5 plays an important role in nervous system development [227,228,229], as well as in angiogenesis, apoptosis, myogenesis, vesicular transport, and senescence in non-neuronal cells [230,231,232,233,234]. In addition, CDK5 dysregulation has been associated with neurodegenerative disorders but its contribution to cancer has been, until recently, overlooked mostly because of the absence of any mutation in tumors. It is now obvious that CDK5 is dysregulated in multiple cancer types, and it needs to receive more attention [235]. 

In GBM, CDK5 is widely overexpressed and can reach up to 83% higher expression level than normal brain tissue [236]. Additionally, Liu et al. showed that CDK5 promoted GBM cell survival, cell migration, and cell invasion by phosphorylating PIKE-A (PI3K enhancer) and by stimulating its GTPase activity which in turn activates nuclear AKT. Furthermore, CDK5 knock-down in glioma stem cells reduced the self-renewal capacity of GSCs both in vitro and in drosophila xenografts [237] and lead to apoptosis in GBM cell models [238]. Owing to all of this, it is unsurprising that CDK5 is emerging as a potential therapeutic target for GBM, and Tau as a substrate of this important kinase suggests that more attention should be given to these two proteins in this context. Especially since Tau can simultaneously bind to CDK5 and the above-mentioned GSK3β kinase [142]. This binding leads to the forming of a complex in which CDK5 phosphorylates Tau at S235 and primes it for phosphorylation of T231 by GSK3β; similarly, CDK5 primes Tau for the sequential phosphorylation of S400 and S396 by GSK3β by phosphorylating Tau at S404. As in the brains of transgenic mice overexpressing CDK5 activity, CDK5 and GSK3β co-localize with hyperphosphorylated Tau [143]. To note, some forms hyperphosphorylated forms of Tau have already been detected in some colon cancer cells such as HCT116 cells [239], and in some prostate cancer cells [14]. 

## 5. Innovative Therapeutic Strategies Targeting Tau Protein for Glioblastoma

**Figure 4:**
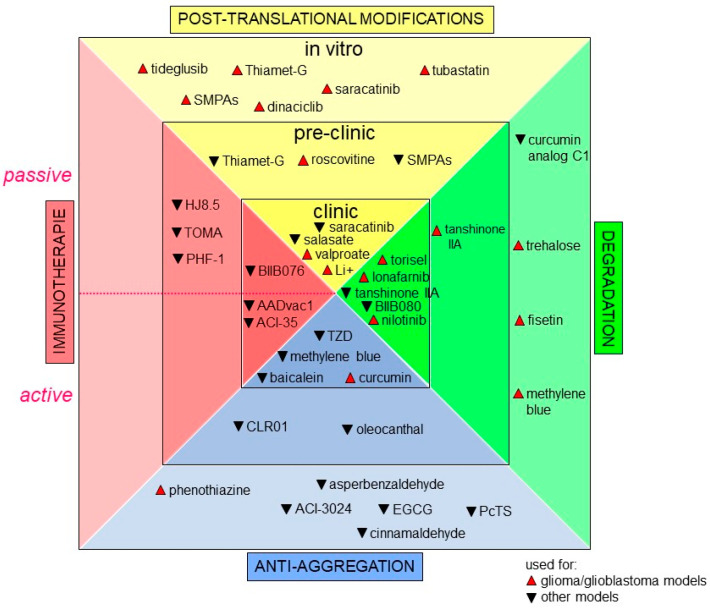
Tau targeting approaches in glioma/ glioblastoma and other pathologies. Tau targeting agents are grouped according to their targeting approach and to their phase of development, in vitro, preclinical, or clinical. Drugs developed for cancer are in red and denoted with upright triangles. Drugs developed for other conditions are in black with an upside triangle. The dashed line in immunotherapies separates the drugs used for passive immunotherapies (upper half) and active immunotherapies (lower half).

Increasing attention is now being paid to precision medicine. Indeed, scientific and medical communities agree to say that healthcare should be finely tuned to each individual based on molecular profiling instead of the “on drug fits all model” currently used [240,241,242]. In GBM, the current standard therapy, using TMZ, only adds 2.5 months to the median survival of patients [22,243]. Hence, it appears crucial to find druggable cancer-associated proteins. Since Tau protein has a major role in many signaling pathways in GBM, it is tempting to think that targeting this protein would be a good promising therapeutic strategy for GBM and maybe even for other cancer types. In GBM, a major challenge for Tau targeting drug discovery is to maximize the probability that the discovered drug, by either biochemical or phenotypic methods, will lead to clinical efficacy and improved disease management [244,245,246,247,248]. This is even more important since many non-cancer cell types such as neurons also express Tau protein. In this regard, these cells can become targets for cytotoxic drugs, and it is crucial to preserve them during therapy. Moreover, key hallmarks of malignancy are clearly not regulated by a single signaling pathway, as we reviewed some of the above. Unfortunately, drugs targeting the elements of essential and core signaling pathways altered in GBM such as EGFR, IDH1, p53, PI3K and more have failed to transfer into efficient clinical agents [249]. One of the reasons behind these failures could be attributed to the significant heterogeneity of GBM tumors [250,251]. Other pitfalls should be overcome with cell-based models that often do not accurately represent the true in vivo situation. For example, the blood–brain barrier remains relatively impermeant to the majority of GBM marker-targeting drugs (see the well-detailed review [252]). Till now, no drug targeting Tau protein has been designed for cancer treatment per se, but many promising compounds developed as a treatment strategy for tauopathies also deserve our attention [253]. We will now summarize approaches consisting of indirect targeting of Tau protein, such as inducing post-translational modifications; or of direct targeting, such as (i) inhibiting Tau aggregation, (ii) active and passive immunotherapies, and (iii) reducing Tau levels in the cell [254,255]. In this chapter, we will talk about some of Tau targeting approaches as an attempt to provide a starting ground for drug development for GBM and other cancers. Figure 4 below summarizes new promising drugs, as well as drug repurposing, all targeting Tau protein.

### 5.1. Playing on Post-Translational Modifications of Tau

In GBM, regulating Tau post-translational modifications could also denote a good strategy to control Tau pathogenicity given that Tau post-translational state of Tau in GBM is studied. The best-described post-modification of Tau is its level of phosphorylation which promotes its aggregation among other pathological defects. In addition to the kinases and the proteins described above, Tau has other interacting proteins that deserve further examination since they were also proven to have an important role in GBM. Among them, we mention the HSP70 chaperone system protein which mediates ubiquitinylation of aberrant Tau species for selective elimination [256,257], the PP2A protein which promotes the dephosphorylation of Tau [258], the HAT/HDAC proteins which can acetylate/deacetylate Tau [259], or even the OGT/OGA proteins which transfer or remove GlcNAc to Tau. Several compounds targeting this set of Tau-regulating proteins are currently being in trial for GBM to examine their potential therapeutic benefit.

#### 5.1.1. Inhibitors of Tau Hyperphosphorylation

##### Src Kinase Inhibitor Saractinib

Apart from the usual EGFR/PI3K/AKT pathway, the tyrosine-protein Src kinase family is currently being targeted. Among promising candidates, we cite saracatinib (AZD0530), a small molecule Src and Fyn inhibitor. Initially developed for AD treatment, Yun and coll. recently evaluated, in vitro and in vivo, the beneficial effect of the combination of saracatinib and radiation on GBM-derived cells U251 [260]. They observed a saracatinib-induced radiosensitization of cells. This was associated with the persistence of radiation-induced γH2AX foci, with no specific cell cycle and mitotic index changes. Interestingly, the authors showed that treatment with saracatinib also blocked the EGFR/PI3K/AKT pathway activation induced by radiation. As the U251 cell model displays a quite high expression of Tau [106], it is tempting to suppose that a reduction in the phosphorylation of Tau induced by the inhibition of the Src pathway (and of the EGFR/PI3K/AKT one) may represent a novel good therapeutic approach of GBMs, though it remains to verify Tau phosphorylation level in these cells.

##### GSK3β Kinase Inhibitors

A second interesting Tau kinase is GSK3β which is overexpressed in GBM as mentioned above. Its suppression in vitro has been shown to induce apoptosis of cancer cells. In this sense, many chemical GSK3β inhibitors have been explored, initially to reverse Tau pathogenicity in AD such as lithium (Li^+^), valproate (VPA), and tideglusib (TDG) (see the in-depth review of [261]). 

Concerning Li^+^, Nowicki and coll. reported by experimental evidence that Li^+^ potently and reversibly blocked glioma cell migration and invasion in all tested cell lines [223]. Indeed, Li^+^ caused the retraction of the long protrusions at the front of the migrating cell, which was fully reversible upon withdrawal of treatment. Moreover, the authors demonstrated that the degree of GSK3β inhibition was inversely correlated with the degree of invasion, suggesting that GSK3β inhibition directly impacts cytoskeleton-regulated migration of glioma cells. In addition, it is important to note that significant effects of Li^+^ on glioma migration were observed at high concentrations of around 5 mM, a dose with severe toxic effects in a clinical context. 

In addition, the clinical effectiveness of VPA in adjuvant GBM treatment has yet to be entirely investigated, and especially due to the lack of randomized prospective phase III trials that show indication of progression-free or overall survival benefit [262,263]. To better understand the interest of VPA in GBM therapy, the work of Hu et al. provides some experimental evidence [264]. Using AD transgenic mice as well as human neuroblastoma SH-SY5Y cells, they determined that VPA inhibited both the activities of GSK3β and CDK5 kinases, and consequently reduced Tau hyperphosphorylation. 

Concerning TDG, no clinical trial is underway to our knowledge to examine the beneficial effects of this GSK3β inhibitor in cancer therapy. However, few in vitro results deserve our attention [265,266]. By combining TDG with X-ray radiation treatment on 2D and 3D cultured GBM-derived U251 and U118 cells, Fares and Abou-Kheir’s teams found reduced cell proliferation, cell viability, and migration in a dose- and time-dependent manner [267]. These results suggest strongly that TDG may serve as a potential adjuvant radiotherapeutic agent to better target GBM tumors. Given our limited progress in improving GBM survival, we suggest that the repositioning of these drugs, initially explored for AD, deserves attention. Therefore, it would be interesting to hold them to clinical efficacy demonstration for GBM. 

##### CDK Inhibitors

CDK inhibitors (CDKi) are developed with great caution. Some of them are in pre-clinical and clinical trials, particularly those targeting CDK5 [268,269,270,271]. In this context, the best-described CDK5 inhibitor is roscovitine (seliciclib), a small molecule that inhibits CDKs through direct competition at the ATP-binding site. It is a broad-range purine inhibitor of CDK5, as well as CDK2/7. Roscovitine is widely used as a biological tool in cell cycle, cancer, apoptosis, and neurobiology studies and even clinical trials using roscovitine have been conducted on patients with cancer [268]. Using the RG2 rat glioma model, Yakisich and coll. found that roscovitine caused a high decrease in glioma cell proliferation coupled with a strong inhibition of DNA synthesis. The cytotoxic activity of roscovitine could have originated from both an early effect on the DNA replication machinery and a late effect on cell cycle progression. Here, it is important to remember that CDK5 inhibition enhances Tau phosphorylation by activating GSK3β. Therefore, it is tempting to think that CDKi-induced Tau phosphorylation could also contribute to defected cell cycle progression. On the other hand, to our knowledge, none of these studies shows a direct correlation between the anti-tumor activity of roscovitine and the expression and/or phosphorylation of Tau, which deserves to be examined. 

#### 5.1.2. Compounds Stimulating Tau Dephosphorylation: Case of PP2A Activation

The serine/threonine protein phosphatase 2A (PP2A) is the most active enzyme in dephosphorylating Tau protein [258]. Therefore, stimulating PP2A activity may represent an alternative strategy to kinase inhibition. In this topic, Sangodkar and coll. reported that improved small-molecule activators of PP2A (SMAPs), synthetic tricyclic sulfonamide, were able to inhibit the growth of KRAS-mutant lung cancer in mice xenografts and mice transgenic models through binding to the PP2A Aα scaffold subunit driving by such activation of the protein [272]. Hence, SMAPs can directly activate PP2A, leading to cell death and lung tumor suppression by inhibiting MAPK signaling pathway. Recently, the same research team demonstrated clearly that the two SMAPs DBK-1154 and DBK-1160 decreased Tau phosphorylation in two cell models [273]. These data reveal that pharmacological PP2A activation may be a novel therapeutic strategy for cancer treatment in general and for GBM therapy in particular.

#### 5.1.3. Compounds Regulating Tau Acetylation/Deacetylation: HAT/HDAC Proteins

Epigenetic regulation of genes plays a key role in numerous disorders. In this process, histone acetylation controls the ease with which transcription factors can access DNA to regulate gene expression. Two classes of enzymes, histone acetyltransferases (HAT) and histone deacetylases (HDAC), control histone acetylation and deacetylation, respectively [274]. In GBM, many HAT mutations such as those of lysine acetyltransferase MOZ and MORF proteins have been reported [275]. To our knowledge, no study in oncology has reported that Tau can be acetylated by HAT proteins. However, an indirect relationship between the two proteins could occur through p53 which has been identified as a MOZ/MORF non-histone protein acetylation substrate [276]. Recently, a clinicians and researchers’ consortium study found that two MOZ and MORF inhibitors, WM-8014 and WM-1119, showed promise for lymphoma therapy [277]. As oligomeric Tau can be a substrate for p53 [214], it should be interesting to deeply examine the beneficial effect of HAT inhibitors as a new therapeutic option in GBM.

An alternative approach for the broad, yet selective, acetylation of Tau protein by HAT is the inhibition of HDAC activity. In the past two decades, in vitro studies have shown significant promise in HDAC inhibitors (HDACis) that could synergize with other compounds in cancer treatment (see the reviews by [278,279]). This could provide a rationale to apply these synergistic ways in GBM as well. Recently, Perez and coll. demonstrated that low concentrations of an HDAC6 inhibitor, vorinostat (SAHA) induced changes in microtubule cytoskeleton properties in U87MG cells [280]. Here, they focused on End-protein 1 (EB1), a MAP stabilizing GTP cap on microtubule plus ends. They found that micromolar concentrations of SAHA caused a strong decrease in EB1 expression. More interestingly, they evidenced that SAHA also induced an increase in tubulin acetylation, a marker of the stabilized microtubule, as well as reduced cell migration. To note, this U87MG cell model exhibits a strong expression level of Tau protein [98,129]. Furthermore, the association of HDAC6 with Tau protein has already been evidenced and it was shown to occur through HDAC6 deacetylase and ZnF UBP domains. The consequence of this association consists of a reduction in HDAC6 deacetylase activity on α-tubulin [259,281]. Moreover, Tau acetylation at lysine residues in KXGS motifs can promote Tau hyperphosphorylation and favors its accumulation [282,283]. As suggested by Honoré’s team [280], low doses of SAHA could represent an interesting therapeutic option for GBM, especially in patients with EB1 and/or Tau overexpressing tumors.

#### 5.1.4. Inhibitors of Tau O-GlcNAcetylation

O-GlcNAcylation refers to the posttranslational modification of O-linkage of N-acetyl-glucosamine (GlcNAc) moieties to serine and threonine residues on proteins. This is regulated by the O-GlcNAc transferase (OGT) and the O-GlcNAcase (OGA), which transfers GlcNAc to and removes GlcNAc from proteins, respectively. The newly emerged OGA selective inhibitor thiamet-G has shown its potential benefit in the therapy of Tau hyperphosphorylation-associated neurodegenerative disorders [284]. In cancers, Ding and coll. reported that thiamet-G significantly sensitized diverse human leukemia cell lines to paclitaxel, with an approximate 10-fold leftward shift of IC50. Interestingly, they observed that paclitaxel combined with thiamet-G resulted in more profound perturbations on microtubule stability than did either one alone. One of the explanations could be that the O-GlcAcetylation of Tau promotes its microtubule-stabilizing function by preventing its hyperphosphorylation [285]. These findings thus suggest that OGA inhibitors such as thiamet-G may serve as a potential adjuvant chemotherapeutic agent for the treatment of GBM.

### 5.2. Reducing Tau Levels in the Cell

The rationale behind reducing Tau quantity in cancer cells stems from the contribution of this protein to tumorigenesis, as exposed above. Two strategies, in addition to antibodies use, were explored to reduce Tau levels, (i) directly inhibiting Tau protein expression with antisense oligonucleotides (ASO), or (ii) inducing Tau clearance. 

#### 5.2.1. Antisense Oligonucleotides Assayed in AD That Could Be Used in GBM

Although there are many ASO designed for AD treatment purposes, only BIIB080 also known as IONIS-MAPTRx or ISIS 814907 has made it into clinical trials for AD as the first and only ASO targeting Tau expression (NCT03186989). This ASO was designed with some modifications to improve its nuclease resistance, increase its cellular uptake, and to induce RNase H-mediated degradation of Tau mRNA as described in a review by the authors [286]. The preclinical study showed that this ASO not only reduces human Tau levels in PS19 mice, but it also decreases Tau mRNA and protein expression in non-human primate Cynomolgus monkeys after intrathecal administration [287]. The primary results of phase I/II clinical trials for AD based on this ASO are to be expected in 2022 (NCT03186989). It could be interesting to explore this approach for cancers, especially in GBM.

#### 5.2.2. Tau Clearance by the Autophagy-Lysosomal System

This strategy consists of stimulating Tau protein degradation mechanisms. In addition to the ubiquitination-proteasome system [288], targeting the autophagy–lysosome system (ALS) could represent an interesting therapeutic avenue [289,290,291,292]. ALS includes three major autophagy pathways, macroautophagy, microautophagy, and chaperone-mediated autophagy (CMA), all leading to lysosome degradation of proteins and cargos [293]. Overall, all modifications of Tau, such as mutation [290,291,292,293,294] and/or post-translational modification such as acetylation [283], impair its correct clearance by this pathway and interferes with global lysosomal functioning. This leads to Tau accumulation inside the cell. Interestingly, in AD and related tauopathies and even in GBM, CMA is dysfunctional [295]. CMA involves Heat shock cognate 70 kDa protein (Hsc70), a member of the Heat shock protein 70 family (Hsp70), that identifies and escorts substrates to the lysosome for degradation [296]. In this way, Jinwal and coll. found that Hsc70 rapidly engages Tau after microtubule destabilization and increases the accumulation of the protein within the cell [297]. Moreover, they showed that cell treatment with methylene blue, well-described to inhibit Hsc70 ATPase activity [290], leads to better Tau degradation.

Hsc70 inhibitors aside, numerous compounds natural or synthetic have been reported to increase Tau clearance in cells such as EA-1 [298], Li^+^ [299], Trehalose [300], the mTor inhibitor temsirolimus (torisel^©^) [301], nilotinib [302], lonafarnib [303], curcumin analog C1 [304], the flavonoid Fisetin [305], Flubendazole (fluvermal^©^) [306,307], Bromhexine [308], tanshinone IIA [309], and other molecules via activation of one or multiple Tau degradation pathways. Some of these cited compounds are already in phase 2 clinical trials (see the review of [310]). 

### 5.3. Therapies Primarily Developed for Tauopathies

#### 5.3.1. Modulation of Tau Aggregation

Likewise, in the regulation of Tau post-translational modifications, the lack of knowledge on the Tau aggregation state in GBM makes it hard to assess the potential efficiency of aggregation inhibitors for treatment. In contrary to neurons, Tau oligomers accumulation in cells was not toxic such as in prostate cancer but they sensitized them to radiotherapy [95]. Alternatively, we can also propose that modulators of Tau aggregation may sequester monomeric Tau making it unavailable for other interactions; therefore, this approach might show some benefit in GBM therapy. Interestingly, Gandini and coll. recently adopted a new strategy that consisted of targeting both Tau aggregation and phosphorylation by GSK3β at the same time and identified some thiazolidinedione derivatives that had this potential in the AD model [311]. Molecules that have dual actions on Tau and major kinases may be the most suitable to be used for GBM.

Regarding the search for new Tau aggregation modulators, the structure–activity studies carried out in the context of neurodegenerative diseases showed that Tau protein is prone to aberrant conformational changes that cause its aggregation and the formation of neurotoxic PHFs. Therefore, these inhibitors can act directly on Tau aggregates dissociation, as well as prevent the formation of Tau aggregates. It is this last propriety that may be a potential therapeutic strategy for GBM tumors. These inhibitors can be grouped into two classes, covalent and non-covalent Tau aggregation inhibitors [312]. The covalent Tau aggregation inhibitors act mainly on monomeric Tau by covalently and directly modifying it, but they can also act on non-monomeric Tau proteins by covalently altering bonds between or within non-monomeric Tau proteins [312]. Examples include many chemical compounds with aldehyde groups such as oleocanthal, cinnamaldehyde, and asperbenzaldehyde, some flavonoids such as baicalein, some molecules that belong to the aminothienopyridazines family, and finally some phenothiazines such as methylene blue. Moreover, non-covalent Tau aggregation inhibitors are structurally more diverse; they can act on different Tau species and have various modes of action which range from transiently interacting with Tau and depressing its aggregation by blocking lysine residues or by sequestering the protein. Examples of this group include: phthalocyanine tetrasulfonate (PcTS; [313]), phenothiazines, triarylmethines, curcumin, and some of the derivatives such as PE859 and some molecular tweezers such as CLR01 among other small or large molecules [314,315].

#### 5.3.2. Immunotherapies against Tau Protein

Tau immunotherapies have advanced from proof-of-concept studies to over a dozen clinical trials for AD and other tauopathies, of which there are ten clinical trials on passive immunotherapy and two on active immunotherapy (see well-detailed reviews in [316,317,318]). Briefly, passive immunotherapy consists of antibody administration which will lead to Tau protein clearance, while active immunotherapy is a vaccine developed against Tau protein to establish immunity in patients. Although we can fairly say that the field is still in its infancy for cancer, this interesting approach exhibits some interesting aspects, as we will discuss below.

##### Passive Immunotherapy

As mentioned above, the purpose of passive Tau immunotherapy is to neutralize pathogenic Tau forms from the brain to reduce its neurotoxicity. Evidently, this type of immunotherapy has mainly been explored in neuropathologies such as AD. To date, several antibodies have been developed to target monomeric or aggregated Tau, phospho-specific Tau, conformational forms of Tau, or a combination of more than one Tau form. As for their mode of action, some antibodies can only act extracellularly where they sequester extracellular Tau and prevent its aggregation or sequester Tau aggregates and promote their phagocytosis by microglia, preventing the propagation of Tauopathy to other neurons [319,320,321]. Other antibodies can act both extracellularly, in a similar manner to the previous ones, and intracellularly by sequestering cytosolic Tau; they promote its degradation by addressing aggregates to the lysosome system [322,323,324]. In the clinic, most of the trials are ongoing and their detailed outcome has yet to be published. Although worth mentioning, the implementation of this therapeutic strategy to GBM may seem implausible. Additionally, and as mentioned before, the lack of knowledge on Tau forms found in GBM makes it hard to predict which antibody may be more suitable for use.

##### Active Immunotherapy

The attractive side of this approach is that vaccines would induce autologous antibody production by patients without generating antidrug antibodies. Nevertheless, vaccines are not without issues themselves, off-target immune response presents a big concern for Tau vaccine development [325]. This means that the choice and the criteria of choice of the Tau fragment to be used as a vaccine should be very well studied. Regardless, if Tau vaccines show to be effective, we can imagine a preventive usage of vaccines for people more susceptible to developing tauopathies. Indeed, active Tau immunotherapy has been exclusively examined in AD therapy with two ongoing clinical trials [317]. The first Tau-directed vaccine tested in clinical trials was AADvac1, developed by Axon Neuroscience SE. These antibodies are expected to prevent Tau protein aggregating, facilitate the removal of Tau aggregates, and prevent the spreading of the pathology, slowing or halting the progression of the disease. The Tau fragment used corresponds to 151–391 residues of the 4R isoform and hence, the antibody recognizes an epitope in the microtubule-binding domain repeat region. The second phospho-Tau-specific vaccine is ACI-35 which was initially developed by AC Immune and later licensed by Janssen. Here, the Tau fragment used corresponds to 393–408 residues in the C-terminal end of the protein. This fragment includes several phosphorylated serine residues (S396/S404) known to be pathologically phosphorylated by GSK3 kinases in AD. In the future, when more research is completed in this context, we can reassess the benefit of using Tau vaccines for GBM.

## 6. Conclusions and Perspective

Through this review, we explained how Tau could be involved in cancer and particularly in GBM. Firstly, we showed how this protein can be implicated in cancer by exploring its role in various cancer hallmarks, and then we discussed some of the pathways highly altered in GBM and how Tau protein can be involved in them. We also mentioned some of the Tau interactors that have demonstrated roles in GBM. Lastly, we summarized some of the innovative therapeutic approaches targeting Tau for the treatment of GBM. Through the evidence presented above, we believe that this area of research is worth exploring and we hope that this review will inspire researchers to investigate the role of Tau protein in this type of cancer.

## 7. Limitations of This Review

One of the clear limitations of this review is the lack of research completed on Tau in GBMs and most of the arguments made are also based on results of research completed on other types of cancer or through the known functions of Tau that can be relevant in this topic.

## Figures and Tables

**Table 1:** Overview of Tau interactions with important effectors, kinases, and transcription factors, involved in altered signaling pathways and their effects on cellular responses in glioma/glioblastoma. The involvement of Tau protein in each signaling pathway is indicated in “Tau status” column, as being “up-stream” or “downstream” of the pathway. n.d: not determined; n.a.: not applicable; ?: pathway, effector, or Tau status not specified in referred studies.

Altered Pathways		Reported Targets	Tau Status	Cell Responses	References
RTK pathway:	EGFR/Ras/MAPK	IDH	n.d.	Survival, proliferation	[65,96,126]
	EGFR/Ras/MAPK	NFκB/TAZ	Upstream	Survival, angiogenesis	[65]
	PI3K/AKT ?	mTOR	Upstream ?	Drug resistance	[127]
	PI3K	-	n.a.	Interaction (in vitro)	[14,128]
	PI3K/AKT	n.d.	Upstream	Migration, adhesion, proliferation	[129]
	PTEN (deletion, mutation)	PI3K/AKT	Upstream	Survival, proliferation	[129,130,131]
	EGFR/PI3K/AKT ?	p53	Upstream ?	Survival	[132,133]
	PI3K/AKT ?	GSK3β, 14-3-3	Downstream	Survival, proliferation	[134,135,136,137,138,139,140,141]
	PI3K/AKT	CDK5, GSK3β	Downstream	Survival, proliferation, migration	[142,143]
SKF pathway:	Src	RTK (PDGFR)	Upstream	Survival, migration	[99]
	Fyn	RTK (EGFR, PDGFR, c-MET) ?	Upstream	Survival, proliferation	[144,145,146]

## Data Availability

Not applicable.
